# Epigenetic and transcriptomic alterations in the ClC-3-deficient mice consuming a normal diet

**DOI:** 10.3389/fcell.2023.1196684

**Published:** 2023-05-23

**Authors:** Zhenghui Jing, Haifeng Zhang, Yunjie Wen, Shiyu Cui, Yuhua Ren, Rong Liu, Sirui Duan, Wenbao Zhao, Lihong Fan

**Affiliations:** ^1^ Department of Pathology of Basic Medicine College, Xi’an Jiaotong University, Xi’an, China; ^2^ Institute of Genetics and Developmental Biology of Translational Medicine Institute, Xi’an Jiaotong University, Xi’an, Shaanxi, China; ^3^ Guangzhou Huayin Medical Laboratory Center Ltd., Guangzhou, Guangdong, China; ^4^ Department of Cardiovascular Medicine, The First Affiliated Hospital of Xi’an Jiaotong University, Xi’an, Shaanxi, China

**Keywords:** glucose metabolism, ClC-3, genetically modified mice, DNA methylation, normal diet

## Abstract

**Introduction:** Metabolic disorders are an important health concern that threatens life and burdens society severely. ClC-3 is a member of the chloride voltage-gated channel family, and ClC-3 deletion improved the phenotypes of dysglycemic metabolism and the impairment of insulin sensitivity. However, the effects of a healthy diet on transcriptome and epigenetics in ClC-3^−/−^ mice were not explained in detail.

**Methods:** Here, we performed transcriptome sequencing and Reduced Representation Bisulfite Sequencing for the liver of 3 weeks old WT and ClC-3^−/−^ mice consuming a normal diet to insight into the epigenetic and transcriptomic alterations of ClC-3 deficient mice.

**Results:** In the present study, we found that ClC-3^−/−^ mice that were younger than 8 weeks old had smaller bodies compared to ClC-3^+/+^ mice with ad libitum self-feeding normal diet, and ClC-3^−/−^ mice that were older than 10 weeks old had a similar body weight. Except for the spleen, lung, and kidney, the average weight of the heart, liver, and brain in ClC-3^−/−^ mice was lower than that in ClC-3^+/+^ mice. TG, TC, HDL, and LDL in fasting ClC-3^−/−^ mice were not significantly different from those in ClC-3^+/+^ mice. Fasting blood glucose in ClC-3^−/−^ mice was lower than that in ClC-3^+/+^ mice; the glucose tolerance test indicated the response to blood glucose increasing for ClC-3^−/−^ mice was torpid, but the efficiency of lowering blood glucose was much higher once started. Transcriptomic sequencing and reduced representation bisulfite sequencing for the liver of unweaned mice indicated that ClC-3 deletion significantly changed transcriptional expression and DNA methylation levels of glucose metabolism-related genes. A total of 92 genes were intersected between DEGs and DMRs-targeted genes, of which Nos3, Pik3r1, Socs1, and Acly were gathered in type II diabetes mellitus, insulin resistance, and metabolic pathways. Moreover, Pik3r1 and Acly expressions were obviously correlated with DNA methylation levels, not Nos3 and Socs1. However, the transcriptional levels of these four genes were not different between ClC-3^−/−^ and ClC-3^+/+^ mice at the age of 12 weeks.

**Discussion:** ClC-3 influenced the methylated modification to regulate glucose metabolism, of which the gene expressions could be driven to change again by a personalized diet-style intervention.

## 1 Introduction

The prevalence of metabolic disorders is steadily rising along with the improvement of living quality. According to the International Diabetes Federation (IDF) Diabetes Atlas, it is estimated that 537 million people have diabetes and 541 million people are suffering from impaired glucose tolerance in 2021 (Sun et al., 2022). The metabolic dysfunction of glucose has been one of the non-negligible risks, threatening human life and curbing social and economic development. Thus, it is urgent to identify efficient molecular targets to rebalance the dysfunction of glucose metabolism.

Epigenetics is a heritable and reversible modification that acts as a mediator of gene–environmental interactions. DNA methylation, an essential epigenetic modification to silence gene expression, has been focused on as a clinical biomarker in cancer (Jin et al., 2009; Payne, 2010), aging (Teschendorff et al., 2010), obesity (Xu et al., 2013; Aslibekyan et al., 2015), cardiovascular events (Hu et al., 2021; Qin et al., 2021), Alzheimer’s disease (Shao et al., 2018), etc. Gathered evidence demonstrated that aberrant DNA methylation was closely related to diabetes. For instance, pancreatic duodenal homeobox 1 (PDX-1) mutations can cause a monogenic form of maturity-onset diabetes of the young, in which the CpG sites in the distal promoter and enhancer regions exhibited significantly increased DNA methylation in the pancreatic islets of type 2 diabetes (T2D) patients (Yang et al., 2012). Furthermore, the genome-wide survey revealed predisposing T2D-related DNA methylation variations in human peripheral blood, including CENTD2, FTO, KCNJ11, TCF7L2, and WFS1 (Toperoff et al., 2012).

ClC-3 chloride channels are widely expressed in most mammalian cells and are primarily localized on cell and organelle membranes. Regarding the important roles in the vasculature’s pathological changes, ClC-3 was considered a promising therapeutic target for cardiovascular diseases (CVDs) (Duan, 2011; Du and Guan, 2015; Niu et al., 2021). It is well known that diabetes, dyslipidemia, and hypertension are CVDs’ most common risk factors. ClC-3 could mediate the processing and release of insulin through the regulation of granular acidification (Deriy et al., 2009; Li et al., 2009). ClC-3 deficiency attenuated hyperglycemia, hyperlipidemia, and insulin resistance in type 2 diabetes mellitus (Huang et al., 2014), and it ameliorated high-fat diet-induced obesity, which promoted the development of glucose and lipid metabolism disorders (Ma et al., 2019). It is supposed that ClC-3 would be a potential target to improve the dysfunction of glucose or lipid metabolism.

ClC-3 silencing remarkably refined glucose and lipid metabolism in animals intaking an abnormal diet, but the underlying mechanisms were not clearly elucidated, especially the changes of metabolism-related gene modification. The existence of a gene is the foundation of its expression, but the expression levels are the results of a life entity responding to the environment. Thus, this study primarily demonstrated the influence of a normal diet on transcriptome and DNA methylation in ClC-3 knockout mice.

## 2 Materials and methods

### 2.1 Animals

ClC-3 knockout mice were obtained from the mating of heterozygous mice, which were produced via the CRISP-cas9 technique (Cyagen, China) and gifted by Doctor Deng from the First Affiliated Hospital of Shen Zhen University. The mice were fed under a 12-h dark/light cycle with *ad libitum* self-feeding in specific pathogen-free conditions of 55% humidity and 22°C. All animal experimental procedures were approved by the Laboratory Animal Administration Committee of Xi’an Jiaotong University (2021-1499).

The wild-type (ClC-3^+/+^), heterozygous (ClC-3^+/−^), and homozygous mice (ClC-3^−/−^) were identified by a polymerase chain reaction following our previous work (Jing et al., 2022). The length of the PCR product for the WT ClC-3 was 769 bp, for which the sequences of primers were TTA​GTG​CTG​GCT​GTG​GCA​TC (F1) and TCC​CAG​AGA​CAA​TGA​GGC​TAA​GG (R). The PCR product of 654 bp was for the mutated ClC-3, and the sequences of primers were TCT​GAT​GGG​GAC​TAA​GTA​TGC​AG (F2) and TCC​CAG​AGA​CAA​TGA​GGC​TAA​GG (R).

### 2.2 Serum and tissue sample collection

The unweaned mice at the age of 3 weeks were anesthetized with 0.03% pentobarbital sodium solution and then euthanized by cervical dislocation; their liver, kidney, lung, heart, spleen, and brain were dissected, photographed, and stored at −80°C. The 4-week-old ClC-3^+/+^ and ClC-3^−/−^ mice were fed a normal diet and weighed weekly. After 8 weeks, all mice were anesthetized with 0.03% pentobarbital sodium solution; the blood obtained from the heart was placed at 4°C for 2 h and then centrifuged at 4,000 rpm for 15 min to harvest serum; the principal parenchymal organs of 12-week-old mice were then dissected, weighed, photographed, and cryopreserved.

### 2.3 Glucose tolerance and blood lipid test

The 12-week-old adult mice were rolled into the glucose tolerance test. After being made to fast for 12 h, the mice were intraperitoneally injected with D-glucose doses of 2 g per kilogram body weight (CAS-50997, Sigma-Aldrich, United States); the glucose of tail blood at 0, 15, 30, 60, 90, and 120 min after injection was measured using a glucometer (Accu-Chek-Guide, Roche, Germany), respectively. The time–concentration curves of blood glucose for ClC-3^+/+^ and ClC-3^−/−^ mice were plotted and standardized according to the fasting blood glucose.

After the 12-week-old mice fasted overnight, the total cholesterol (TC), triglyceride (TG), high-density lipoprotein (HDL), and low-density lipoprotein (LDL) were assayed with an automated biochemical analyzer (BS-800, Mindray, China).

### 2.4 Transcriptomics

The transcriptome of a 3-week-old mouse’s liver was sequenced by the Huayin Health Medical Group Company (Guangzhou, China). In brief, total RNAs were extracted using the TRIzol reagent (cat.265709, Life, United States) following the manufacturer’s instructions. After being qualified and quantified by the Agilent 2100 Bioanalyzer (Agilent, United States) and NanoPhotometer^®^ (Implen, Germany), 1 μg RNA was used to purify mRNA via the VAHTS^®^ mRNA Capture Beads with Oligo dT (cat.N401-01, Vazyme Biotech, China). Subsequently, mRNA was reversely transcripted into the double-strand cDNA with VAHTS^®^ Universal V6 RNA-seq Library Prep Kit (cat.NR604, Vazyme Biotech, China). Then, cDNA was added to an A base and digested with the UDG enzyme. Favorable cDNA fragments were selected for PCR amplification to establish the cDNA library. Finally, 2 × 150 bp paired-end sequencing was performed on a NovaSeq™ 6000 system (Illumina Corporation, United States) under the vendor’s guidance.

Raw reads proceeded to quality control, and then, clean data were obtained. After being blasted with the mouse reference genome (mmu10), the algorithm of RNASeq by Expectation Maximization (RSEM) was adopted to compute the gene expressions, of which the differential expression analysis between ClC-3^+/+^ and ClC-3^−/−^ mice was done with edgeR (Robinson, United States). The genes with |log2Ratio| ≥ 1 and *p*-value <0.05 were regarded as differentially expressed genes (DEGs), which were furtherly explored by the enrichment of Kyoto Encyclopedia of Genes and Genomes (KEGG) pathways.

### 2.5 Quantitative real-time polymerase chain reaction

The total RNA of livers in 12-week-old mice was extracted by TRIzol reagent (#R4801, Magen, China) according to the instruction and quantified with a nanodrop instrument (DeNovix, United States). A total of 1 μg RNA was reverse-transcribed into complementary DNA (cDNA) using a reverse transcription kit (#1137ES60, Yeasen, China). The qPCR was performed using a CFX real-time system (Bio-Rad, United States), and the machine parameters were pre-degeneration at 95°C for 5 min; denaturation at 95°C for 10 s, and annealing and extension at 60°C for 30 s for 40 cycles were performed. The 2^−ΔΔCT^ method was used to calculate the relative expression of genes. GAPDH was used as a reference gene for analyzing target gene expression. The primer sequences used in the study are listed in Table 1.

**TABLE 1:** Primer sequences for qPCR used in the study.

Gene	Forward primer	Reverse primer
GAPDH	CAG​TGG​CAA​AGT​GGA​GAT​TGT​TG	TCG​CTC​CTG​GAA​GAT​GGT​GAT
Nos3	GGT​TGC​AAG​GCT​GCC​AAT​TT	TAA​CTA​CCA​CAG​CCG​GAG​GA
Pik3r1	CTA​TGC​CTG​CTC​CGT​AGT​GG	TCA​TCG​CCT​CTG​TTG​TGC​AT
Acly	GTT​GCG​TTT​GTG​GAC​ATG​CT	ATC​CCA​GGG​GTG​ACG​ATA​CA
Socs1	CGA​GAC​CTT​CGA​CTG​CCT​TT	AGT​CAC​GGA​GTA​CCG​GGT​TA

### 2.6 Reduced representation bisulfite sequencing

Reduced representation bisulfite sequencing (RRBS) was performed to analyze the DNA methylations of liver tissues, which were the same samples being used in transcriptome sequencing. In brief, the genomic DNA was isolated with the QIAamp^®^ Blood and Tissue Kit (cat.51104, Qiagen, Germany). After the quality inspection of DNA, 100 ng DNA was digested with MspI, end-repaired, 3'-dA-tailed, and ligated to 5-methylcytosine-modified adapters. DNAs were treated with bisulfite and then amplified via PCR to establish RRBS libraries, of which the concentration and fragment size were detected by using Agilent 2100 Bioanalyzer and Qubit assay tubes (cat.1604220, Life, United States). Finally, 2 × 150 bp paired-end sequencing was performed on an Illumina NovaSeq™ 6000 system following the vendor’s recommended protocol.

The low-quality reads and sequencing adapters were trimmed from the sequencing raw data to obtain clean data using Trimmomatic software (version 0.40, Germany). DNA methylation information was obtained by blasting clean data with the mouse reference genome (mmu10) using the BSMAP protocol (version 2.90). The differentially methylated regions (DMRs) in the CG context were analyzed with a binary segmentation algorithm combined with the MWU-test and 2D KS-test using metilene software (version 0.2.8, United States). DMR-targeted genes were enriched by the analysis of KEGG pathways.

### 2.7 Conjoint analysis of transcriptomics and DNA methylation

The sample used in methylation sequencing was incised from the corresponding liver tissue in transcriptome sequencing, so the conjoint analysis of these data could be executed. After the DEGs in transcriptomics and DMR-targeted genes were intersected, the mutual genes were analyzed with the enrichment of KEGG pathways. Moreover, the Pearson correlations between the intersected gene expressions and their DNA methylation levels were computed.

### 2.8 Statistical analysis

All data were presented as mean ± standard error. The difference between the two groups was tested by an independent Student’s *t*-test, and a *p*-value less than 0.05 was considered significantly different. All statistical analyses were performed with SigmaPlot software (v12.5, Systat Software Inc., United States).

## 3 Results

### 3.1 The deletion of ClC-3 did not impede the weight gain of ClC-3^−/−^ mouse consuming a normal diet

ClC-3^−/−^ mice with breast milk or high-fat diets gained lower body weight (Jing et al., 2022), indicating the important roles of ClC-3 in energy metabolism. The manifestations were the results of the interactions between the genes and environment, including diet, lifestyle, and emotion. However, there is no idea about the effects of *ad libitum* self-feeding normal diet on the growth of ClC-3^−/−^ mice.

**FIGURE 1:**
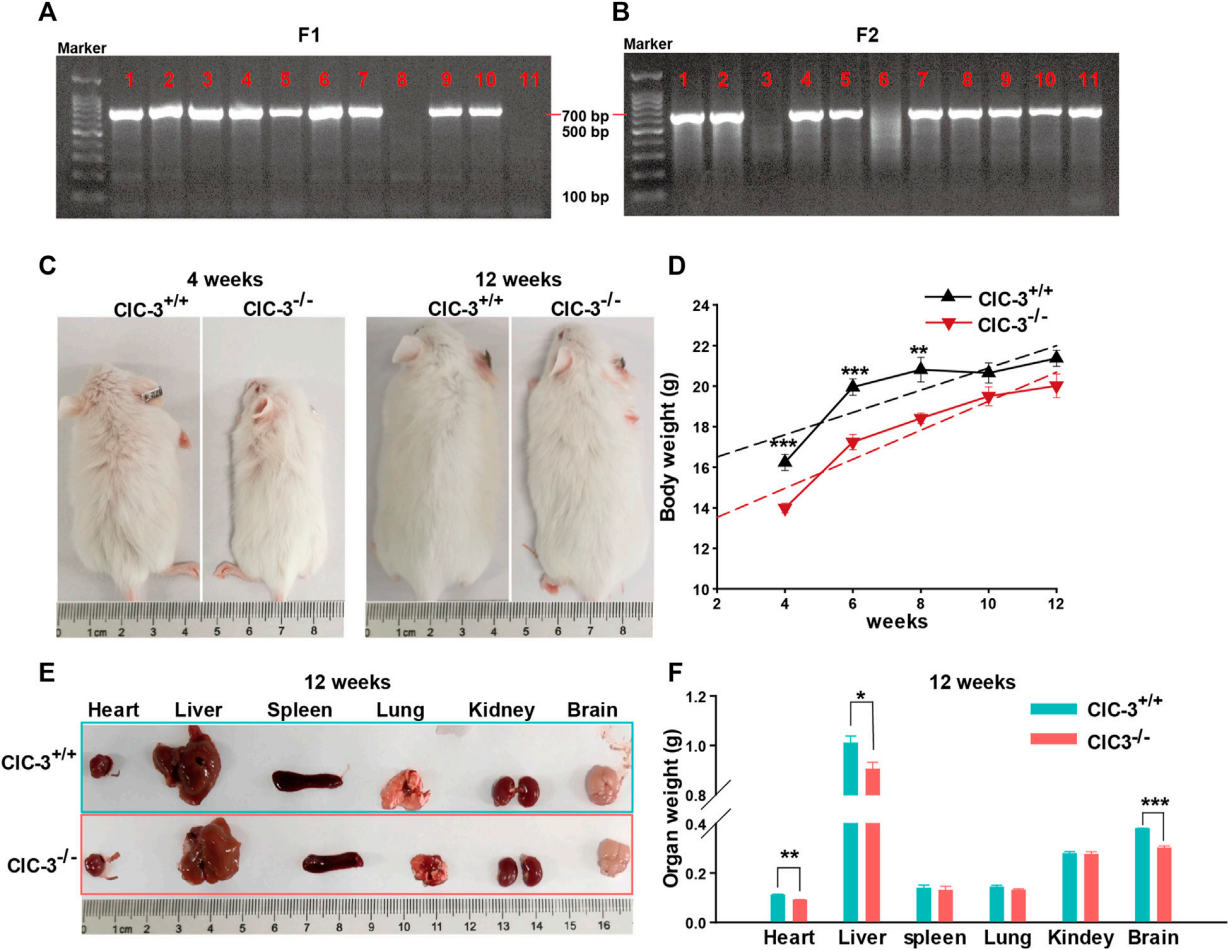
Knockout ClC-3 did not affect the gain of body weight of mice on a normal diet. **(A)** Gel electrophoresis of PCR production amplified by primer F1 and R. **(B)** Gel electrophoresis of PCR production amplified by primer F2 and R. **(C)** Body phenotype of ClC-3^+/+^ and ClC-3^−/−^ mice at 4 and 12 weeks. **(D)** Body weight of ClC-3^+/+^ and ClC-3^−/−^ mice at 4, 6, 8, 10, and 12 weeks (*n* = 7–8). **(E,F)** Typical images and weight of parenchymal organs (heart, liver, spleen, lung, kidney, and brain) in ClC-3^+/+^ and ClC-3^−/−^ mice at 12 weeks (*n* = 7). *, *p* < 0.05; **, *p* < 0.01; and ***, *p* < 0.001.

As shown in Figures 1A, B, DNA was extracted from the tail of 3-week-old mice and amplified by PCR to determine the ClC-3 genotype; there was only one band of 769 bp PCR product for ClC-3^+/+^ mice and one 654 bp band for ClC-3^−/−^ mice, and two bands indicated the heterozygous mice. Compared to 4-week-old ClC-3^+/+^ mice, ClC-3^−/−^ mice were obviously smaller; their average body weight was 13.99 ± 0.24 g. Mice were fed with a normal diet and grown to 10 weeks old, and the average body weights for ClC-3^+/+^ and ClC-3^−/−^ mice were 20.65 ± 0.5 and 19.5 ± 0.47 g, respectively (*n* = 7–8, *p* > 0.05). There was no difference in body weight between ClC-3^+/+^ and ClC-3^−/−^ mice at the 12 weeks (*n* = 7–8, *p* > 0.05, Figures 1C, D). It is suggested that the growth of mice consuming a normal diet was not obviously slowed by the ClC-3 gene deletion.

Moreover, the major parenchymal organs in 12-week-old mice were dissected, photographed, and weighed. As illustrated in Figures 1E, F, there were no differences in the spleen, lung, and kidney weights for ClC-3^+/+^ and ClC-3^−/−^ mice (n = 7, *p* > 0.05), which were not consistent with those observed in the unweaned mice (Jing et al., 2022). The average weight of the heart, liver, or brain in ClC-3^−/−^mice was lower than that in ClC-3^+/+^ mice (*n* = 7, *p* < 0.05); the total weights of the heart, liver, and brain in ClC-3^+/+^ and ClC-3^−/−^ mice only accounted for 7.07% ± 0.17% and 6.58% ± 0.16% of the whole body weights, respectively (*n* = 7, *p* > 0.05). These observations showed that ClC-3^−/−^ mice gained more body weight than ClC-3^+/+^ mice in the same feeding condition, suggesting that the normal diet may be efficiently converted for development and growth in ClC-3^−/−^ mice.

### 3.2 The deletion of ClC-3 partially impaired glucose tolerance in mice consuming a normal diet

The aforementioned details indicated that ClC-3^−/−^ mice could efficiently obtain the needs in a normal diet to gain weight, for which the utilization of lipids and glucose is essential. Thus, the blood lipid and glucose tolerance tests were performed in 12-week-old mice with a normal diet.

**FIGURE 2:**
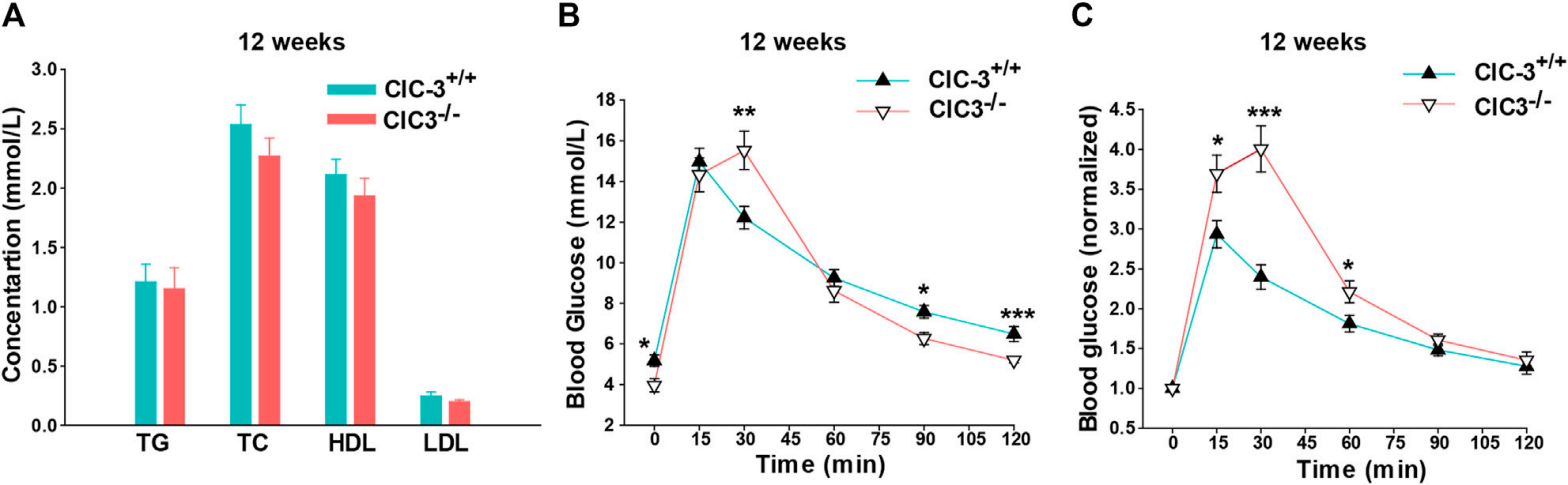
Knockout ClC-3 impaired glucose tolerance in a normal diet. **(A)** The concentration of TC, TG, HDL, and LDL in serum in ClC-3^+/+^ and ClC-3^−/−^ mice at 12 weeks on a normal diet (*n* = 7). **(B)** The concentration of blood glucose at 15, 30, 60, 90, and 120 min time points after being intraperitoneally injected with D-glucose doses of 2 g per kilogram body weight in ClC-3^+/+^ and ClC-3^−/−^ mice consuming a normal diet at 12 weeks (*n* = 7–8). **(C)** Normalized blood glucose according to the fasting blood glucose. *, *p* < 0.05; **, *p* < 0.01; and ***, *p* < 0.001.

As shown in Figure 2A, TG, TC, HDL, and LDL in fasting ClC-3^−/−^ mice were 1.15 ± 0.18, 2.27 ± 0.16, 1.93 ± 0.15, and 0.2 ± 0.02 mM, respectively, which were slightly lower than those in ClC-3^+/+^ mice, but not significant (*n* = 7, *p* > 0.05). Fasting blood glucose (FBG) in ClC-3^+/+^ and ClC-3^−/−^ mice was 5.18 ± 0.28 and 3.97 ± 0.33 mM, respectively (*n* = 7–8, *p* < 0.05). After being intraperitoneally injected with D-glucose doses of 2 g/kg body weight, blood glucose in wide-type mice quickly increased to the peak value of 14.98 ± 0.66 mM at 15 min and then decreased to 6.48 ± 0.37 mM at 120 min; for ClC-3^−/−^ mice, blood glucose increased to 14.33 ± 0.83 mM at 15 min, reached the peak value of 15.53 ± 0.95 mM at 30 min, and then, rapidly decreased to 5.2 ± 0.13 at 120 min (Figure 2B, vs. values in ClC-3^+/+^ mice at the same time, *p* < 0.05). Blood glucose values were normalized according to the autologous FBG to eliminate the FBG difference between ClC-3^+/+^ and ClC-3^−/−^ mice. As illustrated in Figure 2C, after the intraperitoneal injection of D-glucose for 15 min, the increased folds of blood glucose in ClC-3^+/+^ and ClC-3^−/−^ mice were 2.93 ± 0.18 and 3.69 ± 0.23, respectively (*p* < 0.05); for 30 min, the blood glucose-increased folds in ClC-3^+/+^ and ClC-3^−/−^ mice were 2.4 ± 0.15 and 4.0 ± 0.29, respectively (*p* < 0.001); for 90 and 120 min, there were no differences of blood glucose-increased folds between ClC-3^+/+^ and ClC-3^−/−^ mice (*p* > 0.05).

The response to blood glucose increasing for ClC-3^−/−^ mice was torpid, but the efficiency of lowering blood glucose was much higher once started. It is suggested that the deletion of ClC-3 partially impaired glucose tolerance in mice with normal dietary intake.

### 3.3 The deletion of ClC-3 significantly affected the transcriptional expression of genes in glucolipid metabolism-related pathways

The liver, as one of the major organs, is responsible for glycolipid metabolism (Bechmann et al., 2012; Ye et al., 2017), which is crucial for development and growth. Thus, the transcriptomes of the liver in 3-week-old ClC-3^+/+^ and ClC-3^−/−^ mice were sequenced to investigate the roles of ClC-3 in the glucose metabolism of mice with a normal diet.

**FIGURE 3:**
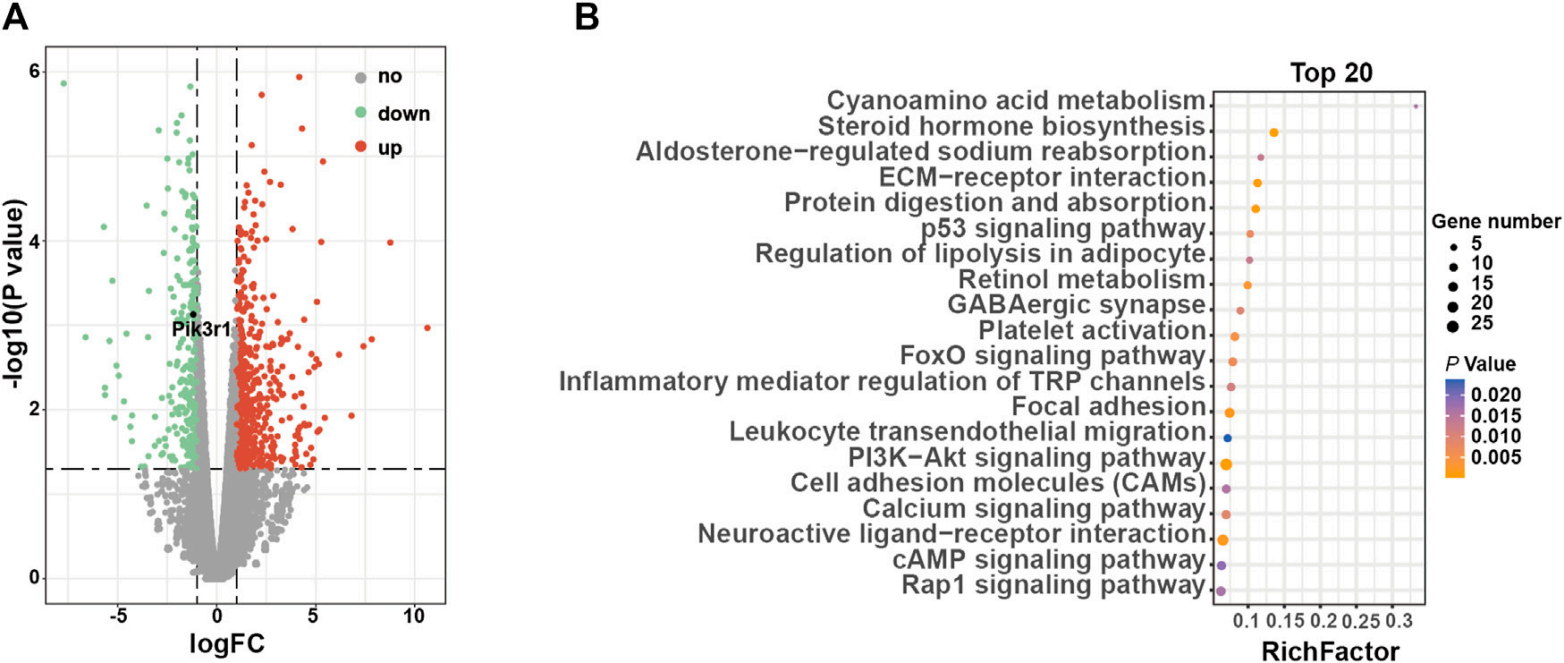
ClC-3 deletion significantly changed the transcriptional expression of glucose metabolism-related genes in unweaned mice. **(A)** Volcano plot of differentially expressed genes in the livers between 3-week-old ClC-3^+/+^ and ClC-3^−/−^ mice (ClC-3^−/−^ v. s. ClC-3^+/+^ mice, *n* = 4–5). **(B)** Top 20 KEGG enrichment pathways of DEGs.

Compared to 3-week-old ClC-3^+/+^ mice, 874 DEGs in ClC-3^−/−^ mice were identified, including 589 upregulated genes and 285 downregulated genes (Figure 3A, *n* = 4–5, *p* < 0.05). Among them, Pik3r1 was marked as a positive control gene, which was reported by another study (Jingxuan et al., 2022). Then, all DEGs were analyzed by the enrichment of KEGG pathways. As illustrated in Figure 3B; Supplementary Table S1, the glucose and lipid metabolism-related pathways were significantly clustered (*p* < 0.05), including steroid hormone biosynthesis, the PI3K-Akt signaling pathway, and the regulation of lipolysis in adipocytes. Moreover, the enriched pathways of amino acid metabolism were ranked in the top 20 (*p* < 0.05).

The aforementioned details suggested that ClC-3-deletion-caused impairment of glucose tolerance may result from the transcriptional changes of glucose metabolism-related genes.

### 3.4 The deletion of ClC-3 changed the levels of DNA methylation in ClC-3^−/−^ mice liver

ClC-3 proteins were expressed in the nucleus (Wang et al., 2000; Mao et al., 2012; Zhang et al., 2013a; Liang et al., 2014) and influenced gene expression (Zhang et al., 2013b). However, the roles of nuclear ClC-3 were not elucidated until now. The aforementioned details showed that hundreds of genes were up- or downregulated by ClC-3 deletion, suggesting that the regulation of gene expression was one of the roles of ClC-3 proteins in the nucleus. DNA methylation is the heritable and reversible controller of gene expression. Then, we observed the effects of DNA methylation on glucose metabolism-related genes in ClC-3^−/−^ mice livers.

**FIGURE 4:**
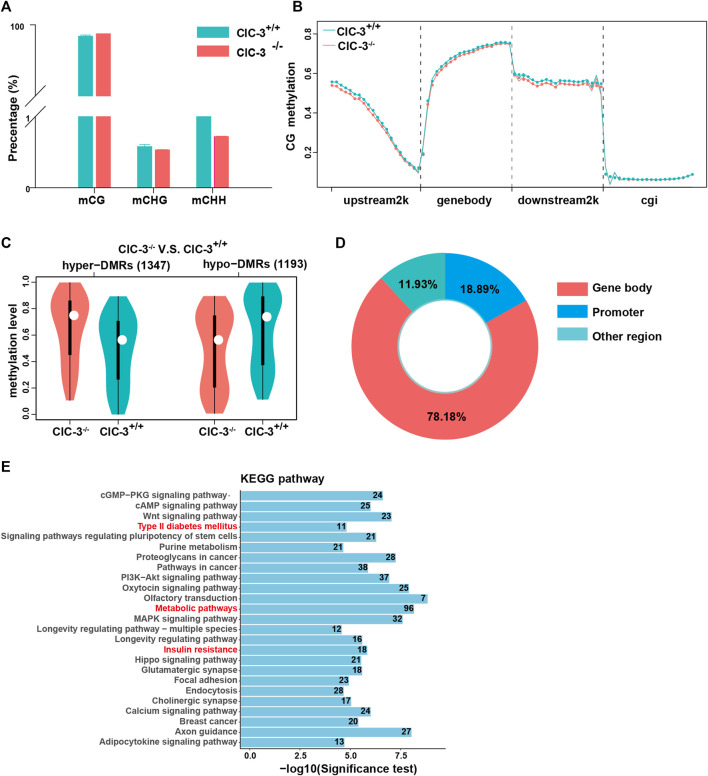
Knockout ClC-3 significantly affected the DNA methylation level of genes in the liver of 3-week-old mice. **(A)** Percentage of methylated cytosines in different contexts (CG, CHG, and CHH) in the liver of 3-week-old ClC-3^+/+^ and ClC-3^−/−^ mice (*n* = 4–5). **(B)** Distribution of methylated cytosines in gene functional elements in ClC-3^+/+^ and ClC-3^−/−^ mice (upstream2K, upstream 2000 bp of the transcription start sites; gene-body; downstream2K, downstream 2000 bp of transcription termination sites; and cgi, CpG island). **(C)** The average methylation level of DMRs between 3-week-old ClC-3^+/+^ and ClC-3^−/−^ mice. **(D)** Distribution of DMRs in the CG context in gene functional elements. **(E)** KEGG enrichment of DMR-anchored genes.

The levels of DNA methylation in the 3-week-old mice livers were analyzed by RRBS. As illustrated in Figure 4A, over 98% of methylated cytosines occurred in a CG context, and nearly 2% of methylated cytosines were detected in CHH and CHG contexts. Then, the methylated cytosine sites in the CG context were further explored. Between ClC-3^+/+^ and ClC-3^−/−^ mice of CG contexts, methylated cytosine sites in the transcription start site’s upstream 2000 bp, gene-body, and the transcription termination site’s downstream 2000 bp were different, but they were not different in the CpG islands (Figure 4B). Additionally, a total of 2,540 differentially methylated regions were found; compared to ClC-3^+/+^ mice, there were 1,347 upregulated DMRs and 1,193 downregulated DMRs in ClC-3^−/−^ mice (Figure 4C); 78.18% of DMRs were overlapped in the gene-body region, and 18.89% of DMRs were located in the promoter of genes (Figure 4D). The genes targeted by DMRs in the CG context were enriched by the analysis of KEGG pathways, the top 25 pathways of which are presented in Figure 4E. The glucose metabolism-associated pathways were significantly enriched, such as type II diabetes mellitus, insulin resistance, metabolic pathways, and citrate cycle. Additionally, the DMR-targeted genes, such as Nos3, Pik3r1, Socs1, and Acly, were identified and involved in these pathways.

These indicated that ClC-3 deletion could change the DNA methylation levels of genes related to glucose metabolism in unweaned mice.

### 3.5 ClC-3 was involved in the glucose metabolism-related gene expression via DNA methylation

DNA methylation is an important pattern to regulate gene expression, but the levels of gene expression are not always in correspondence with the levels of methylation. Then, the relationships between DMR-anchored genes and DEGs were further explored.

**FIGURE 5:**
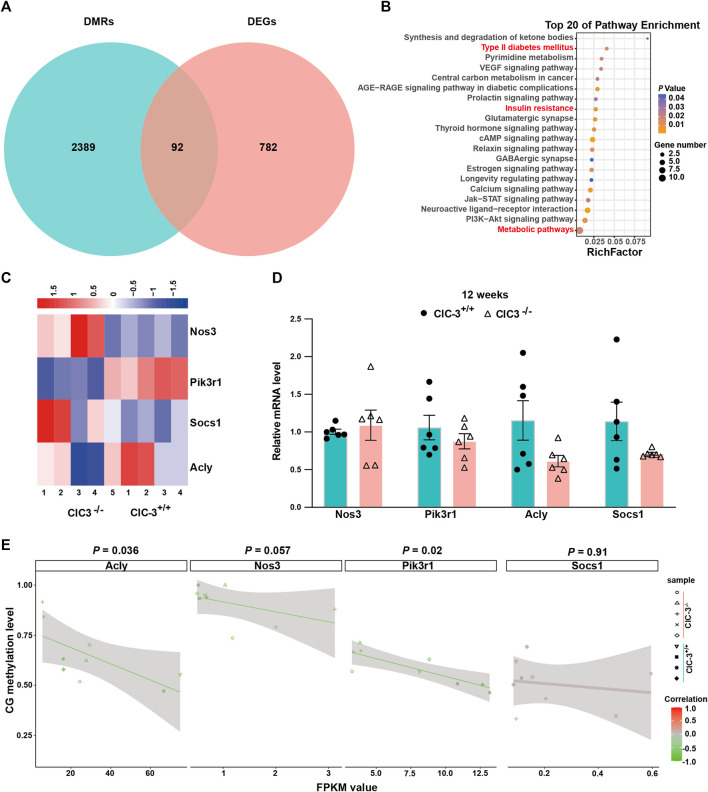
Transcriptional alterations of glucose metabolism-related genes were associated with CpG DNA methylation. **(A)** The intersection between DMR-anchored genes and DEGs in the liver of 3-week-old ClC-3^+/+^ and ClC-3^−/−^ mice. **(B)** KEGG pathway enrichment of intersection genes between DMRs-anchored genes and DEGs. **(C)** Expression heatmap of DEGs (Nos3, Acly, Pik3r1, and Socs1) related to glucose metabolism between 3-week-old ClC-3^+/+^ and ClC-3^−/−^ mice. **(D)** Transcriptional expression of glucose metabolism-related genes (Nos3, Acly, Pik3r1, and Socs1) of 12-week-old ClC-3^+/+^ and ClC-3^−/−^ mice did not alter (qPCR, *n* = 6). **(E)** Correlation of glucose metabolism-related genes between their DNA methylation level and gene expression level.

As shown in Figure 5A, there were 92 overlapped genes between DMR-anchored genes and DEGs. These genes were enriched by the analysis of KEGG pathways, the glucose metabolism-related pathways of which were listed in the top 20, such as type II diabetes mellitus, insulin resistance, and metabolic pathways (Figure 5B, *p* < 0.05). The correlations of overlapped genes between gene expression and DNA methylation were analyzed. There were 14 genes whose expressions were negatively correlated with the methylation levels in gene-body (*p* < 0.05); the expression of five genes was positively correlated with the methylation levels in the gene-body (*p* < 0.05); Onecut1 was the only gene whose expression was positively correlated with the methylation levels in the gene-body’s upstream 2K regions (*p* < 0.05, Supplementary Table S2). These indicated that ClC-3 deletion affected the expressions of specific genes, which were closely associated with DNA methylation in unweaned mice.

Nos3, Pik3r1, Socs1, and Acly, as the overlapped genes, were enriched in the glucose metabolism-associated pathways. Both Socs1 and Pik3r1 were enriched in the PI3K-Akt and insulin signaling pathways. In the unweaned ClC-3^−/−^ mice, the transcriptional levels of Socs1 and Pik3r1 were, respectively, upregulated and downregulated (*p* < 0.05), which could inhibit the insulin receptor-mediated glycogen synthesis. Acly and Nos3 were enriched in the citrate cycle, insulin resistance, and PI3K-Akt signaling pathways. Iin the unweaned ClC-3^−/−^ mice, the transcriptional level of Nos3 was upregulated (*p* < 0.05), which could block the transduction of insulin receptor signaling; Acly was downregulated (*p* < 0.05), which inhibited the breakdown of citric acid to decrease lipid synthesis and increase energy supply (Figure 5C). Additionally, the expressions of Nos3, Pik3r1, Socs1, and Acly were assayed in adult mice with normal dietary consumption; as illustrated in Figure 5D, there were no differences in these four genes’ expressions between ClC-3^+/+^ and ClC-3^−/−^ mice (*n* = 6, *p* > 0.05).

Furthermore, the Pearson correlations between these genes’ expressions and their DNA methylation levels were calculated. As illustrated in Figure 5E, the FPKM values of Pik3r1 and Acly were negatively correlated with their DNA methylation levels, and the Pearson values for Pik3r1 and Acly were −0.75 and −0.7, respectively (*n* = 4–5, *p* < 0.05); the Pearson values for Nos3 and Socs1 were −0.65 and 0.04, respectively (*n* = 4–5, *p* > 0.05). These suggested that DNA methylation was one of the ways for ClC-3 to regulate the expressions of glucose metabolism-associated genes.

## 4 Discussion

The development of metabolic disease is a complex progress involving multiple factors, such as diet (Zinöcker and Lindseth, 2018), genetics (Barroso and McCarthy, 2019), gut microbiome (Turnbaugh et al., 2006; Marchesi et al., 2016), and age (Lee and Park, 2020). Glucose metabolism is the central reaction of all biological metabolic processes, and its dysfunction will lead to the emergence of obesity, diabetes, and cardiovascular diseases. Thus, it is crucial to rebalance glucose metabolism for the control of metabolic diseases.

In addition to participating in proliferation, migration, apoptosis, and drug transport (Zhang et al., 2006; Cuddapah et al., 2012; Zhang et al., 2013b; Ganapathi et al., 2013; Zhou et al., 2018; Chen et al., 2019; Feng et al., 2021), ClC-3 was verified to actively regulate the glycolipid metabolism. ClC-3 deletion would improve the phenotypes of dysglycemic metabolism and the impairment of insulin sensitivity in mice with an abnormal diet (Deriy et al., 2009; Li et al., 2009; Huang et al., 2014; Ma et al., 2019). These improvements were the comprehensive results of the changes in numerous genes’ expressions affected by ClC-3 deletion.

Gene expression is always the result of gene–environmental interaction, of which DNA methylation, histone modification, and noncoding RNAs are key mediators. A small quantity of ClC-3 proteins localized in the nucleus (Wang et al., 2000; Mao et al., 2012; Zhang et al., 2013a; Liang et al., 2014) would provide a possible manner to participate in the gene expression. However, there was no idea about the effects of ClC-3 deletion on the changes in DNA methylation in mice with a normal diet.

We previously reported that the unweaned ClC-3^−/−^ mice had lower body weights and smaller parenchymal organs (Jing et al., 2022). The present study showed that 4-, 6-, and 8-week-old ClC-3^−/−^ mice with *ad libitum* self-feeding normal diet had lower body weights than the same aged ClC-3^+/+^ mice, which is consistent with another report (Dickerson et al., 2002). ClC-3^−/−^ mice aged 10 and 12 weeks had similar body weights to the wild-type mice, but the heart, liver, and brain weights in 12-week-old ClC-3^−/−^ mice were still lower (Figure 1). Disruption of ClC-3 leads to a loss of the hippocampus in 7-month-old mice (Stobrawa et al., 2001). This was not evidenced in mice younger than 7 months, but ClC-3 deletion slowed the brain growth of adult mice, not the unweaned mice (Jing et al., 2022). These indicated that the inhibition of growth and organ development by ClC-3 deletion may not be invariable along with the increase of age in ClC-3^−/−^ mice with a normal diet.

Glycolipid metabolism is vitally important for growth and organ development. In contrast with that of ClC-3^−/−^ mice consuming a high-fat diet (Ma et al., 2019), there were no significant differences in blood lipids between ClC-3^−/−^ and ClC-3^+/+^ mice with a normal diet at the age of 12 weeks; intraperitoneal glucose tolerance test demonstrated that ClC-3 deletion delayed the response to blood glucose increasing and enhanced the efficiency of lowering blood glucose once started (Figure 2). ClC-3^−/−^ mice had a partial impairment of glucose tolerance in the condition of *ad libitum* self-feeding normal diet, which may be associated with the inhibition of insulin secretion by ClC-3 knocking out (Barg et al., 2001; Deriy et al., 2009; Li et al., 2009; Niu et al., 2021). This finding appeared to be inconsistent with the improvement of glucose tolerance impairment in ClC-3^−/−^ mice with obesity or diabetes (Huang et al., 2014; Ma et al., 2019). Of course, the dietary difference among these mice was an inevitable factor that must be considered, which would have a profound impact on the expression of metabolic genes.

One of the most important organs for regulating glycolipid metabolism is the liver (Bechmann et al., 2012; Ye et al., 2017). Our previous and present studies showed that the livers in the unweaned (Jing et al., 2022) and normal diet-consuming ClC-3^−/−^ mice were all smaller. The liver may be one of the determinants contributing to the ClC-3^+/+^ mice phenotypes, especially glucose metabolism in the condition of normal diet intake, whose underlying mechanisms would be explored by the transcriptomic and epigenetic alterations. ClC-3 deletion caused the transcripted changes of 874 genes, a part of which was enriched in the glucose and lipid metabolism-related pathways, including steroid hormone biosynthesis, the PI3K-Akt signaling pathway, and regulation of lipolysis in adipocyte (Figure 3); 2540 DMR-anchored genes were partially clustered in the glucose metabolism-associated pathways, such as type II diabetes mellitus, insulin resistance, metabolic pathways, and citrate cycle (Figure 4). It is implied that the ClC-3 deletion-associated genes in the control of methylation were vital actors in regulating glucose metabolism.

The intersected genes between DEG- and DMR-targeted genes were gathered in type II diabetes mellitus, insulin resistance, and metabolic pathways, which referred to Nos3, Pik3r1, Socs1, and Acly (Figure 5). The upregulated Nos3, Socs1, and downregulated Pik3r1 inhibited the transduction of insulin receptor signaling, being associated with insulin resistance; the downregulated Acly inhibited the breakdown of citric acid, increasing energy production (Zhang et al., 2014; Piñeros Alvarez et al., 2017; Kempinska-Podhorodecka et al., 2019). There were only Pik3r1 and Acly, whose transcription levels were closely related to DNA methylation levels, implying that the methylated modification was not the only way for ClC-3 to participate in the gene expression. However, the specific expressions of these genes were crucial factors to bring out a petite unweaned ClC-3^−/−^ mouse. Interestingly, it is faster to gain body weight for adult ClC-3^−/−^ mice consuming a normal diet. The answers may be found from the unexpected results that there were no differences in Nos3, Pik3r1, Socs1, and Acly transcriptional levels between ClC-3^−/−^ and ClC-3^+/+^ mice. It suggested that diet types could drive the change of gene expression again in ClC-3-deficient mice.

In conclusion, ClC-3 was involved in glucose metabolism and associated with the methylated modification to regulate the transcriptions of glucose metabolism-related genes, whose expressions could be driven to change again by a personalized diet-style intervention. Therefore, we proposed that ClC-3 could be a potential target to rebalance the dysfunction of glucose or lipid metabolism caused by an unhealthy diet.

## Data Availability

The data presented in the study are deposited in the NCBI repository, accession numbers are PRJNA956595 and PRJNA956309.
